# Lipoprotein(a) is associated with necrotic core progression of non-culprit coronary lesions in statin-treated patients with angina pectoris

**DOI:** 10.1186/1476-511X-13-59

**Published:** 2014-04-01

**Authors:** Tsuyoshi Nozue, Shingo Yamamoto, Shinichi Tohyama, Kazuki Fukui, Shigeo Umezawa, Yuko Onishi, Tomoyuki Kunishima, Akira Sato, Toshihiro Nozato, Shogo Miyake, Youichi Takeyama, Yoshihiro Morino, Takao Yamauchi, Toshiya Muramatsu, Kiyoshi Hibi, Mitsuyasu Terashima, Ichiro Michishita

**Affiliations:** 1 Division of Cardiology, Department of Internal Medicine, Yokohama Sakae Kyosai Hospital, 132 Katsura-cho, Sakae-ku, Yokohama 247-8581, Japan; 2 Department of Cardiology, Tsurumi Nishiguchi Hospital, Yokohama, Japan; 3 Department of Cardiology, Yokohama Seamen’s Insurance Hospital, Yokohama, Japan; 4 Department of Cardiology, Kanagawa Cardiovascular and Respiratory Center, Yokohama, Japan; 5 Department of Cardiology, Hiratsuka Kyosai Hospital, Hiratsuka, Japan; 6 Fourth Department of Internal Medicine, Mizonokuchi Hospital, Teikyo University School of Medicine, Kawasaki, Japan; 7 Cardiovascular Center, Yokosuka Kyosai Hospital, Yokosuka, Japan; 8 Department of Cardiology, National Hospital Organization, Disaster Medical Center, Tokyo, Japan; 9 Department of Cardiology, Ebina General Hospital, Ebina, Japan; 10 Division of Cardiology, Showa University Fujigaoka Rehabilitation Hospital, Yokohama, Japan; 11 Department of Cardiology, Tokai University School of Medicine, Isehara, Japan; 12 Department of Cardiology, Tokyo Women’s Medical University, Tokyo, Japan; 13 Department of Cardiology, Saiseikai Yokohama City Eastern Hospital, Yokohama, Japan; 14 Division of Cardiology, Yokohama City University Medical Center, Yokohama, Japan; 15 Cardiovascular Imaging Center, Toyohashi, Japan

**Keywords:** Lipoprotein (a), Necrotic core, Statin, Virtual histology intravascular ultrasound

## Abstract

**Background:**

Statin therapy results in regression and stabilization of coronary artery plaques, and reduces the incidence of coronary artery disease. However, statin therapy does not effectively halt the accumulation of necrotic core in all patients. The purpose of the present study was to identify the predictors associated with necrotic core progression during statin therapy.

**Methods:**

Coronary atherosclerosis in non-culprit lesions was evaluated using virtual histology intravascular ultrasound at baseline and 8 months after statin therapy. One hundred nineteen patients were divided into 2 groups based on necrotic core progression or regression during an 8-month follow-up period.

**Results:**

Patients with necrotic core progression had higher serum lipoprotein(a) [Lp(a)] levels than patients with regression at baseline (16 mg/dL vs. 12 mg/dL, p = 0.02) and at the 8-month follow-up (17 mg/dL vs. 10 mg/dL, p = 0.006). Patients with necrotic core progression had a higher fibro-fatty plaque volume (1.28 mm^3^/mm vs. 0.73 mm^3^/mm, p = 0.002), and less necrotic core (0.56 mm^3^/mm vs. 1.04 mm^3^/mm, p < 0.0001) and dense calcium (0.35 mm^3^/mm vs. 0.56 mm^3^/mm, p = 0.006) plaque volumes at baseline than patients with regression. Multivariate logistic regression analysis showed that Lp(a) was a significant independent predictor associated with necrotic core progression during statin therapy (odds ratio [OR]: 3.514; 95% confidence interval [CI]: 1.338-9.228; p = 0.01).

**Conclusions:**

Serum Lp(a) is independently associated with necrotic core progression in statin-treated patients with angina pectoris.

## Background

Intensive lipid-lowering therapy with statins reduces the risk of coronary events [1]. Although the mechanisms by which statins provide cardiovascular benefits are not clearly understood, regression and stabilization of coronary artery plaques are presumed to play important roles in this effect [2,3]. Virtual histology (VH)-intravascular ultrasound (IVUS) uses spectral analysis of radiofrequency ultrasound backscatter signals, which allows the identification of 4 different types of atherosclerotic plaque components: fibrous, fibro-fatty, necrotic core, and dense calcium [4]. The rupture of a thin fibrous cap over the necrotic core followed by thrombus formation is the most prevalent cause of acute coronary syndrome [5]. Increasing necrotic core size is associated with plaque instability, and its volume is also associated with distal embolization after percutaneous coronary intervention (PCI) [6]. Statin therapy changes the composition of coronary artery plaques evaluated by using VH-IVUS [7-10]; however, the accumulation of necrotic core could not be halted with statin therapy in all patients. Therefore, in the present study, we compared clinical characteristics, serum lipid profiles, and grayscale and VH-IVUS parameters between patients with necrotic core progression and those with regression to identify predictors associated with necrotic core progression during statin therapy.

## Methods

### Patients and study design

The present study is a post-hoc subanalysis of the Treatment With Statin on Atheroma Regression Evaluated by Intravascular Ultrasound With Virtual Histology (TRUTH) trial. The TRUTH study was a prospective, open-labeled, randomized, multicenter trial performed at 11 Japanese centers to evaluate the effects of 8 months of treatment with pitavastatin versus pravastatin on coronary atherosclerosis using VH-IVUS [7]. Briefly, 164 patients with angina pectoris were randomized to either pitavastatin (4 mg/day, intensive lipid-lowering) or pravastatin (20 mg/day, moderate lipid-lowering) therapy after successful PCI performed under VH-IVUS guidance. None of the participants were taking a statin or other lipid-lowering drugs at the time of study enrollment. Follow-up IVUS examination was performed after 8 months of statin therapy.

The patients were included in the present study if they had measurable IVUS-detected lesions at both enrollment and the 8-month follow-up. Forty-five patients were excluded because of the following reason: withdrew consent in 3 patients, lost to follow-up in 7 patients, IVUS images were not obtained or not analyzable in 28 patients, and adverse events in 7 patients [7]. A total of 119 patients were divided into 2 groups: necrotic core progression or regression. An instance of necrotic core progression was defined as necrotic core volume at follow-up – necrotic core volume at baseline ≥ 0. Necrotic core regression was defined as necrotic core volume at follow-up – necrotic core volume at baseline < 0.

The TRUTH study was conducted in accordance with the Declaration of Helsinki and with the approval of the ethical committees of the 11 participating institutions. Each patient enrolled in the study provided written informed consent.

### IVUS examination and analysis

The details of the IVUS procedure have been documented elsewhere [7]. Briefly, after PCI of the culprit lesion, angiographic lesions that showed no significant stenosis on the coronary angiogram (diameter stenosis <50%) were examined using IVUS. An IVUS catheter (Eagle Eye Gold; Volcano Corporation, San Diego, California) was used, and a motorized pullback device was used to withdraw the transducer at 0.5 mm/s. During pullback, grayscale IVUS was recorded, and raw radiofrequency data were captured at the top of the R wave using a commercially available IVUS console (IVG3; Volcano Corporation). After 8 months of statin therapy, the IVUS examination was repeated in the same coronary artery, using the same type of IVUS catheter that had been used at baseline.

All baseline and follow-up IVUS core laboratory analyses were performed by an independent and experienced investigator (M.T.) in a blinded manner. Before IVUS analysis, baseline and follow-up IVUS images were reviewed side-by-side on a display, and the distal and proximal ends of the target segment were identified on the basis of the presence of reproducible anatomical landmarks such as the side branch, vein, and stent edge. Plaques close to the PCI site (<5 mm) were excluded because mechanical interventions affected atheroma measurements. Quantitative IVUS grayscale analysis was performed according to the guidelines of the American College of Cardiology and European Society of Cardiology [11]. Manual contour detection of the lumen and external elastic membrane (EEM) was performed for each frame. The EEM volume and lumen volume were calculated, and difference between the 2 values was defined as plaque volume. All volumetric data were divided by lesion length to obtain a volume index. VH-IVUS data analysis was based on calculation of grayscale border contour, and relative and absolute amounts of different coronary artery plaque components were measured using IVUSLab version 2.2 (Volcano Corporation). Fibrous tissue was marked in green, fibro-fatty in yellow, dense calcium in white, and necrotic core in red on the VH-IVUS image [4].

### Blood sampling and measurement of blood parameters

Blood samples were obtained after an overnight fast at baseline and at the 8-month follow-up. The levels of serum lipid and high-sensitivity C-reactive protein (hs-CRP) were measured at a central clinical laboratory (SRL Inc., Tokyo). Serum total cholesterol, low-density lipoprotein cholesterol (LDL-C), high-density lipoprotein cholesterol (HDL-C), and triglycerides levels were measured by standard enzymatic methods. Serum oxidized low-density lipoprotein (LDL) levels were measured by an enzyme immunoassay [12]. Serum lipoprotein(a) [Lp(a)] levels were measured by a latex agglutination turbidimetric immunoassay using the commercially available Lp(a)-LATEX (Sekisui Medical Co., Ltd., Tokyo) with an autoanalyzer (JCA-BM8040; JEOL Ltd., Tokyo). Lp(a) particles vary widely in size, with the size heterogeneity related primarily to the different sizes of the apo(a) isoform [13]; however, this method is not affected by apo(a) isoform variation [14]. The intra- and interassay coefficients of variation of Lp(a) were <5.0%, respectively. Serum small dense LDL levels were measured by a homogeneous assay (Denka Seiken Co., Ltd., Tokyo) [15].

### Statistical analysis

Statistical analysis was performed using StatView version 5.0 (SAS Institute, Cary, North Carolina). Results are expressed as mean ± SD, or as median (range). Differences in continuous variables between the 2 groups were compared using Student’s unpaired *t* tests when variables showed a normal distribution, and Mann–Whitney U tests when the variables were not normally distributed. Categorical variables between the 2 groups were compared using chi-square tests or Fisher’s exact tests. Univariate and multivariate logistic regression analyses were performed to assess predictors associated with necrotic core progression during statin therapy. The variables with a p value < 0.1 on univariate analysis were entered into multivariate models. Lp(a) and hs-CRP concentrations were converted to the logarithm for univariate and multivariate logistic regression analyses. Statistical significance was set at p < 0.05.

## Results

### Patients’ characteristics and laboratory results

The baseline characteristics of the subjects are listed in Table 1. Seventy-four patients (62%) were categorized as having necrotic core progression and the remaining 45 (38%) as having regression. There were no significant differences in age, gender, frequency of hypertension and diabetes mellitus, or medications between the 2 groups. However, frequencies of pitavastatin treatment (55% vs. 38%, p = 0.06) and unstable angina pectoris (36% vs. 20%, p = 0.05) tended to be higher in patients with necrotic core progression.

**Table 1:** Baseline characteristics of subjects

	**NC progression**	**NC regression**	**p value**
	**(n = 74)**	**(n = 45)**	
Age (years)	66 ± 10	67 ± 10	0.56
Men	63 (85%)	36 (80%)	0.47
Body mass index (kg/m^2^)	24.7 ± 3.7	24.0 ± 2.8	0.28
Treatment allocation			0.06
Pitavastatin	41 (55%)	17 (38%)	
Pravastatin	33 (45%)	28 (62%)	
Status of coronary artery desease			0.05
Stable angina pectoris	47 (64%)	36 (80%)	
Unstable angina pectoris	27 (36%)	9 (20%)	
Target coronary artery			0.51
Left anterior desending	41 (55%)	26 (58%)	
Left circumflex	2 (3%)	3 (7%)	
Right	31 (42%)	16 (36%)	
Type of stent			0.3
Bare metal stent	15 (20%)	5 (11%)	
Drug-eluting stent	59 (80%)	40 (89%)	
Hypertension	44 (59%)	31 (69%)	0.3
Diabetes mellitus	30 (41%)	20 (44%)	0.68
Medications			
Aspirin	72 (97%)	45 (100%)	0.7
Thienopyridines	73 (99%)	45 (100%)	>0.99
ACE-Is or ARBs	37 (50%)	24 (53%)	0.72
*β* blockers	8 (11%)	5 (11%)	>0.99
Calcium channel blockers	35 (47%)	25 (56%)	0.38
Insulin	5 (7%)	6 (13%)	0.38
Follow-up duration (days)	224 ± 34	231 ± 41	0.31

Data are expressed as mean ± SD or as number (percentage).

NC, necrotic core; ACE-Is, angiotensin-converting enzyme inhibitors; ARBs, angiotensin reporter blockers.

Risk factor control at baseline and at the 8-month follow-up is shown in Table 2. Serum levels of LDL-C, HDL-C, hs-CRP, and small dense LDL, at baseline and at the 8-month follow-up, did not differ between the 2 groups. However, patients with necrotic core progression had higher serum Lp(a) levels than patients with regression at baseline (16 mg/dL vs. 12 mg/dL, p = 0.02) and at the 8-month follow-up (17 mg/dL vs. 10 mg/dL, p = 0.006). Furthermore, oxidized LDL levels at baseline tended to be higher in patients with necrotic core progression (13 U/mL vs. 10 U/mL, p = 0.08). Percentage changes in these parameters did not differ between the 2 groups.

**Table 2:** Risk factor control at baseline and at the 8-month follow-up

	**Baseline**			**8-month follow-up**		
	**NC progression**	**NC regression**	**p value**	**NC progression**	**NC regression**	**p value**
	**(n = 74)**	**(n = 45)**		**(n = 74)**	**(n = 45)**	
TC (mg/dL)	208 ± 35	200 ± 37	0.22	159 ± 26	157 ± 32	0.68
% change				−22 ± 12	−21 ± 13	0.46
LDL-C (mg/dL)	133 ± 31	129 ± 32	0.48	85 ± 22	85 ± 29	0.91
% change				−36 ± 14	−34 ± 18	0.48
Triglycerides (mg/dL)	130 ± 71	132 ± 55	0.92	122 ± 67	108 ± 57	0.26
% change				1 ± 46	−14 ± 37	0.07
HDL-C (mg/dL)	47 ± 12	45 ± 10	0.3	51 ± 13	51 ± 13	0.86
% change				10 ± 25	13 ± 23	0.54
Hs-CRP (ng/mL)	4750 (103 to 88900)	3300 (54 to 76800)	0.57	654 (52 to 26200)	582 (78 to 23300)	0.98
% change				−40 ± 113	−45 ± 73	0.79
Small dense LDL (mg/dL)	26 ± 14	26 ± 13	0.96	18 ± 8	20 ± 10	0.45
% change				−15 ± 52	−19 ± 32	0.76
Oxidized LDL (U/mL)	13 ± 9	10 ± 7	0.08	11 ± 8	9 ± 9	0.18
% change				−3 ± 43	11 ± 166	0.52
Lipoprotein(a) (mg/dL)	16 (3 to 47)	12 (1 to 43)	0.02	17 (1 to 118)	10 (1 to 60)	0.006
% change				26 ± 53	12 ± 49	0.16

Data are expressed as mean ± SD or median (range).

NC, necrotic core; TC, total cholesterol; LDL-C, low-density lipoprotein cholesterol; HDL-C, high-density lipoprotein cholesterol; Hs-CRP, high-sensitivity C-reactive protein; LDL, low density lipoprotein.

### Grayscale and VH-IVUS analysis

The parameters evaluated using grayscale and VH-IVUS are listed in Table 3. The EEM volume index, plaque volume index, and lumen volume index did not differ between the 2 groups. No significant differences were observed in percentage changes in these parameters. However, patients with necrotic core progression had higher fibro-fatty plaque volume and less necrotic core and dense calcium plaque volumes at baseline than patients with regression. Furthermore, changes in each of the 4 plaque component significantly differed between the 2 groups.

**Table 3:** Parameters evaluated using grayscale and virtual histology intravascular ultrasound

	**Baseline**			**8-month follow-up**		
	**NC progression**	**NC regression**	**p value**	**NC progression**	**NC regression**	**p value**
	**(n = 74)**	**(n = 45)**		**(n = 74)**	**(n = 45)**	
EEM volume index (mm^3^/mm)	16.45 ± 5.59	16.18 ± 4.83	0.79	16.09 ± 5.52	16.11 ± 5.02	0.98
% change				−2.1 ± 5.1	−0.5 ± 6.4	0.12
Plaque volume index (mm^3^/mm)	9.11 ± 3.62	8.66 ± 2.73	0.47	8.91 ± 3.37	8.58 ± 2.96	0.58
% change				−1.4 ± 8.5	−1.0 ± 10.1	0.84
Lumen volume index (mm^3^/mm)	7.34 ± 2.58	7.52 ± 2.66	0.71	7.17 ± 2.62	7.54 ± 2.72	0.47
% change				−1.9 ± 11.2	0.5 ± 12.5	0.28
Percent atheroma volume (%)	55.1 ± 7.2	53.6 ± 6.6	0.27	55.3 ± 6.5	53.3 ± 7.5	0.12
Nominal change (%)				0.2 ± 4.0	−0.4 ± 4.4	0.47
Fibrous volume index (mm^3^/mm)	3.50 ± 2.01	2.95 ± 1.44	0.11	3.17 ± 1.74	3.08 ± 1.52	0.78
Change (mm^3^/mm)				−0.34 ± 0.74	0.13 ± 0.84	0.002
FF volume index (mm^3^/mm)	1.28 ± 1.08	0.73 ± 0.57	0.002	0.77 ± 0.68	0.91 ± 0.83	0.33
Change (mm^3^/mm)				-0.51 ± 0.67	0.18 ± 0.53	<0.0001
NC volume index (mm^3^/mm)	0.56 ± 0.41	1.04 ± 0.64	<0.0001	0.99 ± 0.63	0.67 ± 0.44	0.003
Change (mm^3^/mm)				0.43 ± 0.40	−0.37 ± 0.39	<0.0001
DC volume index (mm^3^/mm)	0.35 ± 0.33	0.56 ± 0.50	0.006	0.55 ± 0.48	0.54 ± 0.54	0.92
Change (mm^3^/mm)				0.20 ± 0.27	−0.02 ± 0.31	<0.0001
Average length (mm)	24.7 ± 15.9	24.3 ± 13.3	0.88	24.9 ± 16.1	24.2 ± 13.1	0.81

Data are expressed as mean ± SD.

NC, necrotic core; EEM, external elastic membrane; FF, fibro-fatty; DC, dense calcium.

### Predictors of necrotic core progression

Univariate logistic regression analyses showed that Lp(a) was significantly associated with necrotic core progression during statin therapy whereas pravastatin use or unstable angina pectoris trended (Table 4). Multivariate logistic regression analysis showed that Lp(a) was a significant independent predictor associated with necrotic core progression during statin therapy (odds ratio [OR]: 3.514; 95% confidence interval [CI]: 1.338-9.228; p = 0.01).

**Table 4:** Predictors of necrotic core progression

	**Univariate**			**Multivariate**		
	**OR**	**95% CI**	**p value**	**OR**	**95% CI**	**p value**
Age	0.989	0.953–1.026	0.56			
Male gender	1.432	0.542–3.783	0.47			
Smoking	0.642	0.266–1.548	0.32			
Pravastatin	0.489	0.229–1.042	0.06	0.525	0.233–1.184	0.12
Unstable angina pectoris	2.298	0.962–5.487	0.06	1.934	0.768–4.869	0.16
Hypertension	0.662	0.303–1.450	0.3			
Diabetes mellitus	0.852	0.403–1.803	0.68			
TC	1.003	0.990–1.016	0.68			
LDL-C	0.999	0.984–1.014	0.91			
Triglycerides	1.004	0.997–1.010	0.26			
HDL-C	1.003	0.973–1.033	0.86			
Log (hs-CRP)	0.939	0.501–1.757	0.84			
Small dense LDL	0.983	0.941–1.027	0.45			
Oxidized LDL	1.040	0.979–1.103	0.2			
Log [lipoprotein(a)]	3.664	1.423–9.435	0.01	3.514	1.338–9.228	0.01

OR, odds ratio; CI, confidence interval; TC, total cholesterol; LDL-C, low density lipoprotein cholesterol; HDL-C, high-density lipoprotein cholesterol; hs-CRP, high-sensitivity C-reactive protein, LDL, low-density lipoprotein.

**Figure 1:**
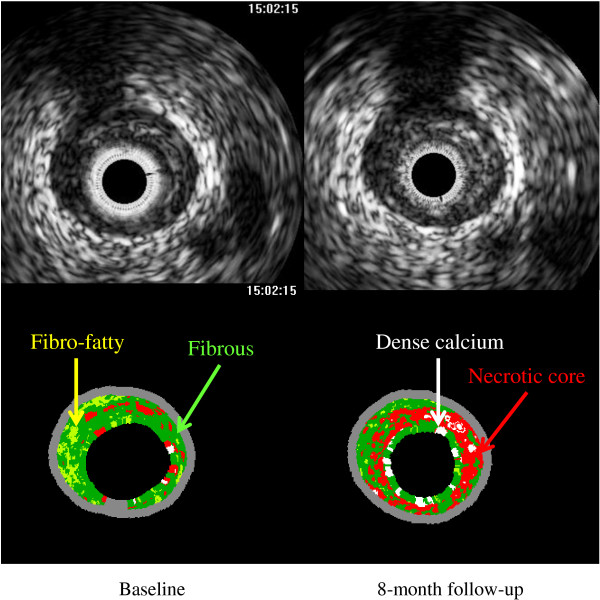
**Representative intravascular ultrasound images of necrotic core progression.** Fibrous areas were marked in green, fibro-fatty in yellow, dense calcium in white, and necrotic core in red on the virtual histology intravascular ultrasound image. This case was a 71-year-old male patient with unstable angina pectoris treated with pravastatin. His baseline serum lipoprotein(a) level was 47 mg/dL. A greater increase in the necrotic core area was observed at the 8-month follow-up.

**Figure 2:**
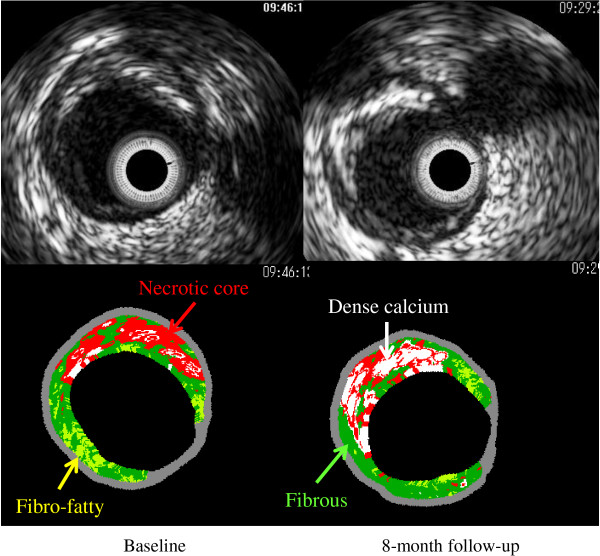
**Representative intravascular ultrasound images of necrotic core regression.** This case was a 78-year-old male patient with stable angina pectoris treated with pravastatin. His baseline serum lipoprotein(a) level was 3 mg/dL. A greater reduction of the necrotic core area was observed at the 8-month follow-up.

The representative IVUS images are shown in Figures 1 and 2. Figure 1 shows IVUS images of a 71-year-old male patient with unstable angina pectoris whose baseline serum Lp(a) level was 47 mg/dL. A greater increase in the necrotic core area was observed at the 8-month follow-up. Figure 2 shows IVUS images of a 78-year-old male patient with stable angina pectoris whose baseline serum Lp(a) level was 3 mg/dL. A greater reduction of the necrotic core area was observed at the 8-month follow-up.

## Discussion

The major findings of the present study are as follows: (1) Lp(a) was a significant independent predictor associated with necrotic core progression during statin therapy; (2) patients with necrotic core progression had higher fibro-fatty plaque volume and less necrotic core and dense calcium plaque volumes at baseline than patients with regression; and (3) 62% of patients treated with statin showed necrotic core progression during an 8-month follow-up period.

Lp(a) is a recognized risk factor for cardiovascular disease [16,17]. Mechanisms that account for the association of Lp(a) with cardiovascular disease have been considered to be its structural similarity to plasminogen which could cause competition with plasminogen activators [18]; intraluminal thrombus formation may also cause a proliferative response of the vascular wall. A recent report supports its role as a prothrombotic factor in that Lp(a) appears to be significantly associated with carotid artery occlusion but not plaque size [19]. However, Helgadottir et al. reported a stronger association of Lp(a) with atherosclerosis than with thrombosis [20]. Furthermore, Lp(a) is associated with plaque progression, as indirectly assessed based on lumen changes on angiography [21,22]. Although there are few data directly demonstrating a relationship between serum Lp(a) and coronary atherosclerosis, serum Lp(a) is an independent predictor of plaque progression [23]. A consensus paper issued Lp(a) as a causal risk factor for cardiovascular disease, and recommended screening for Lp(a) in patients judged to be at high risk for future cardiovascular disease [24]. Indeed, Lp(a) is associated with future cardiovascular events [25] and an adverse prognosis [26]. Our study, therefore, supports the likelihood that Lp(a) is a good predictor of future cardiovascular events, because serum Lp(a) is associated with necrotic core progression during statin therapy.

The underlying mechanisms by which Lp(a) contributes to the pathogenesis of atherosclerosis are not fully understood [27]. The pathophysiologic role of Lp(a) in atherosclerotic disease progression is explained by the accumulation of Lp(a) in the vessel wall, and its ability to promote cholesterol accumulation in macrophages forming foam cells and subsequent fatty streaks [28,29]. Kiechl et al. reported a strong correlation between circulating Lp(a) levels and oxidized phospholipid/apolipoprotein B complex which suggested that Lp(a) transported the proinflammatory burden of oxidized phospholipids [30]. The atherogenicity of Lp(a) may be mediated in part by associated proinflammatory oxidized phospholipids, and is associated with angiographically documented coronary artery disease [31]. A recent study has documented that Lp(a) and oxidized phospholipids mediated macrophage apoptosis [32]. Since macrophage apoptosis is a key component of plaque vulnerability, these phenomena may lead to necrotic core progression as observed in this study. Thus, Lp(a) may mediate antifibrinolytic, proinflammatory, and proapoptotic effects, including those potentiated by its content of oxidized phospholipids [27].

There is no consensus regarding the effects of statins on the necrotic core component [7-10]. Thus, the accumulation of necrotic core could not be halted with statin therapy in all patients. These differences may have occurred because of lipophilic and hydrophilic statins because lipophilic pitavastatin promotes oxidant stress-induced apoptosis of vascular smooth muscle cells, whereas hydrophilic pravastatin does not [7,33]. In addition, statin therapy could not halt the increase in plaque vulnerability in patients with unstable angina pectoris [34]. The differences of types of statin and subject population could account for these discrepancies. Indeed, pitavastatin use and unstable angina pectoris was associated with necrotic core progression on univariate regression analyses.

Additional important results of the present study were that 62% of patients treated with statin had necrotic core progression during an 8-month follow-up period and this may associate with future cardiovascular events as previously reported [35]. In addition, plaques with greater fibro-fatty and less necrotic core component were prone to necrotic core progression during statin therapy. These results are consistent with previous reports that the fibro-fatty component is a reversible step of atherosclerosis [7,8,36]. As observed in this study, Lp(a) concentration is not influenced by statin therapy [37]. However, Lp(a) is also the dominant risk factor for the severity and progression of coronary artery disease in patients treated with statins [38], our results support these reports. Intensive lipid-lowering therapy with statins reduces the risk of coronary events [1]; however, there is a residual risk. This implies a role for Lp(a) in being one of the potential etiologies for the presence of residual cardiovascular risk, and increases the priority for investigation of Lp(a) as a potential therapeutic target. Niacin was able to reduce serum Lp(a) levels [39,40], and niacin added to simvastatin decreased carotid intima-media thickness [41], whereas no incremental clinical benefit was observed from the addition of niacin to statin therapy [42]. Further studies are necessary to investigate better therapeutic options in statin-treated patients with high Lp(a) levels.

This study has several limitations. First, it was a post-hoc subanalysis and the results of this study may be biased because all subjects were treated with statins. However, this study demonstrate that the residual risk of cardiovascular events during statin therapy can be explained in part by Lp(a). Second, although we excluded patients with angiographically apparent thrombi, an intramural thrombus might have influenced the study results. In addition, the IVUS examination was performed in only the non-culprit lesions in the culprit vessel. Mechanical interventions might have affected the atheroma measurement. Furthermore, although VH-IVUS is a promising new diagnostic tool for visual interpretation of plaque characterization, it is not a substitute for pathologic sampling. Nair et al. reported that dividing a plaque into 4 distinct tissue components was difficult because of the presence of amorphous overlapping zones [33]. Third, although Lp(a) appears to act an acute-phase reactant under some situation [43,44], we could not analyze the subjects with stable and unstable angina pectoris separately because of statistical power. Finally, this study is limited by the relatively small number of patients.

## Conclusions

Serum Lp(a) level is independently associated with necrotic core progression in statin-treated patients with angina pectoris. In particular, the data suggest considerable incremental value in using Lp(a) for predicting cardiovascular risk during statin therapy.

## Abbreviations

CI: Confidence interval; EEM: External elastic membrane; HDL-C: High-density lipoprotein cholesterol; hs-CRP: High-sensitivity C-reactive protein; IVUS: Intravascular ultrasound; LDL: Low-density lipoprotein; LDL-C: Low-density lipoprotein cholesterol; Lp(a): Lipoprotein(a); OR: Odds ratio; PCI: Percutaneous coronary intervention; TRUTH: Treatment with statin on atheroma regression evaluated by intravascular ultrasound with virtual histology; VH: Virtual histology.

## Competing interests

The authors declare that they have no competing interests.

## Authors’ contributions

TN contributed to the study design, analysis and interpretation of data, and manuscript preparation. SY, ST, KF, SU, YO, TK, AS, TN, SM, YT, YM, TY and TM contributed to acquisition of data. KH contributed to the study design. MT contributed to the analysis of the data. IM contributed to the study design and managed the study. All authors read and approved the final manuscript.
